# Home range of three turtle species in Central Yucatan. A comparative study

**DOI:** 10.1186/s12862-024-02258-7

**Published:** 2024-05-29

**Authors:** Ivette Enríquez-Mercado, Taggert G. Butterfield, Rafael Aguilar-Romero, Rodrigo Macip-Ríos

**Affiliations:** 1 Escuela Nacional de Estudios Superiores, Unidad Morelia, Universidad Nacional Autónoma de México, Antigua Carretera a Pátzcuaro No. 8701, Ex Hacienda San José la Huerta, 58190 Morelia, Michoacán, México; 2 Estudiantes Conservando la Naturaleza A.C, Álamos, 85760 Sonora México

**Keywords:** Overlap, Movements, Seasons, *Kinosternon*, *Terrapene*, *Rhinoclemmys*

## Abstract

Home range is a fundamental characteristic of an animal natural history. The study of home range provides information on the sites where organisms forage for food, find shelter, or locate mates. Home range size and shape can change throughout the lifespan of an organism, during the year, or across seasons, driven by resource availability and the basic needs for each organism. For freshwater and semi-aquatic turtles, home range is greatly affected by water availability, humidity, and temperature throughout the year, nevertheless demographic factors such age and sex are also important determinants of home range size. In this study we estimated home range and dispersal movements for *Kinosternon creaseri, Terrapene yucatana*, and *Rhinoclemmys areolata* in a semi-tropical dry forest in central Yucatán. For a two-year period, turtles were surveyed using hoop traps and visual encounters. Twenty-one individuals (5–8 per species) were equipped with radio transmitters to track them across the landscape. Distances between relocations and home range were compared across species seasons, sex, and interactions of these variables. Monthly average movements were positively correlated with rain in the three species studied. Home range of *R. areolata* was larger than those of *K. creaseri* and *T. yucatana.* Home range of the three studied species were larger during the wet season. Home range overlap index within same species individuals was higher during the rainy than dry season, but overall overlap is low between and within species.

**Supplementary Information:**

The online version contains supplementary material available at 10.1186/s12862-024-02258-7.

## Significant Statement

Comparative studies of home range in sympatric organisms can shed light on how organisms partition resources to co-exist, and identify the habitat needs for each species. We compared the home range and movement of three sympatric turtle species on the Yucatan Peninsula, Mexico, a habitat characterized as semideciduous dry forest. Home range and movements differ among species and seasons. Turtles move larges distances and have larger home ranges during the rainy season, while their home range shrank during the dry season, when some species estivate, while others occupy permanent water bodies. Our results show that home range is related to season and water availability for the species studied, which used different of microhabitats, with *Kinosternon creaseri* used sartenejas and rock shelters, while *Rhinoclemmys areolate* used wooded debris and leaf litter microhabitats, finally, *Terrapene yucatana* was observed in rocky and woody debris microhabitats.

**Supplementary Information:**

The online version contains supplementary material available at 10.1186/s12862-024-02258-7.

## Background

Movement is a key behavioral trait of animals. One way to study movements is by documenting the home range size [1], which is defined as the area used for all the activities such as foraging, courtship, or taking refuge [2, 3]. A general assumption in statistical modeling is that all habitat and diet resources across a landscape are equally distributed and accessible for all animals [4]. However, resources and landscapes are heterogenous and home range, movement, and how organisms interact with their habitat can be heavily impacted by biotic and abiotic factors [5]. Biotic factors that can affect home range include age, sex, diet, reproductive stage food availability, and food preference [4, 6–9]. Abiotic factors that can impact home range include temperature, precipitation, water availability, landscape heterogeny, and the availability of microhabitats [7, 10]. Additional extrinsic factors, like habitat quality, water quality, or deforestation can significantly impact how an organism utilizes space [11].

When similar species occur in sympatry and use similar resources it can lead to the evolution of distinct morphology, behavior, and physiology, facilitating competitive exclusion [12]. Studying how potentially competing species use their habitat can lead to an understanding of how animals evolved to co-exist [13]. Home ranges among related species could be similar, however, depending on the availability of suitable resources species may tend to avoid each other and use distinct habitats to avoid competition for similar resources [14, 15]. On the other hand, home range may not matter, and species may find ways to avoid resource overlap while sharing the same habitat by having differences in activity or habitat use [16, 17].

Home range overlap and comparisons in turtle communities have been done, but the large majority have focused on turtle communities that are dominated by aquatic turtles. Pérez-Santiagosa et al. [18] for example compared the home range of three aquatic species (including an exotic species) in southern Spain, finding variation between species and small amounts of overlap. Haas [19] compared the home range of a native turtle species with an introduced turtle species in the Upper Niagara River, with some overlap but larger home range of the native species. Vogt and Gúzman-Gúzman [20] also documented the differentiated use of habitat of a three-species turtle community in Mexico. Only recently have studies aimed to understand resource partitioning in turtle communities that include terrestrial turtles [21, 22], but they did not estimate home range or compare microhabitat use and activity patterns among sympatric species.

Turtles’ communities vary in richness and diversity. In some hotspots like the Mississippi river basin or in southeast Asia, they are exceptionally diverse, while in other sites diversity tends to be lower compared to other reptiles’ assemblages [23, 24]. In Mexico, turtle communities range from 7 to 2 species per site [25]. In this study, we focus on a community of three species *Kinosternon creaseri*, *Rhinoclemmys areolata*, and *Terrapene yucatana* in the Puuc hill region of Yucatán. The three studied species represent three different lineages (Kinosternidae, Geomydidae, and Emydidae) and have different body sizes, with *K. creaseri* being the smallest species with an average carapace length (CL) of 109 mm for males and CL = 102 mm for females, followed by *T. yucatana* CL = 155 mm of maximum carapace length. The largest and heaviest of the three species was *R. areolata* CL = 188 mm for males and 178 mm for females. Also, these species have different habits. *K. creaseri* is semiaquatic, with frequent incursions on land and an omnivorous diet (but skewed to feed on insects). *Rhinoclemmys areolata* is mainly terrestrial, with occasional incursions into water, and have an omnivorous diet (with high plant matter). Finally, *T. yucatana*, is terrestrial and has an omnivorous diet [25–27].

Based on a meta-analysis of home range size across turtles, Slavenko et al. [28] found that larger turtles tend to have larger home range sizes. Thus, our first prediction is that *R. areolata* have a larger home range sizes than the other two species followed by *T. yucatana*, and *K. creaseri*. Second, based on studies of movement in other species in tropical dry forests [17], we predicted home range size and movements would be strongly correlated to dry and wet seasons, with movements increasing in the wet season.

## Methods

*Study area*. – The study was conducted in a Biocultural Reserve, located in the center region of the Yucatan Peninsula, at approx. 25 km southwest of Oxkutzcab (precise georeferenced and reserve name is absent due the potential poaching activity in the area). The study site is in the Puuc region, which is part of the Sierra de Ticul, the only mountain range in the Yucatan Peninsula. The study area is covered by tropical semi-deciduous to perennial forest [29]. The climate is warm and sub-humid with rains during summer (Aw). The first rains tend to arrive in late May or early June, and the last heavy rains occur until October [30], with a maximum yearly rainfall total of 1202 mm. For analysis of dry and wet seasons, we define the dry season between the months of November – May, and the wet season between June – October. The study area and the Yucatan peninsula does not have any flowing rivers, and the landscape at our study site is characterized by dispersed permanent and seasonal limestone ponds that are called *haltunes* (Mayan) or *sartenejas* (Spanish). There is wide variation in canopy height and tree diameter across the Puuc hills, with two extremes of macrohabitat, short forest and tall forest. Short forest occurs in disturbed areas and areas with rocky soils trees tend to be smaller. Tall forest is characterized by loamy soils with taller and wider trees. There were three prominent limestone pools on the study site that differed in size and depth, sarteneja A was 4 m deep and 15 m wide, sarteneja B was 2 m deep and 10 m wide, and sarteneja C was 1.5 m deep and 6 m wide. These sartenejas were an average of 936 m (linear) apart from each other. In addition to the large sartenejas, there were many smaller ponds that ranged from 15 cm wide to a meter wide that we did not record.

*Sampling protocol*. – Field surveys for turtles were conducted at the study site in 2018 and 2019 and select adult individuals were equipped with radio transmitters. To capture turtles that were making terrestrial movements, foot surveys were conducted in potential turtle habitats to locate individuals. To trap *K. creaseri* in sartenejas we used hoop nets baited with sardines. We used 10–15 g Holohil R1-2B transmitters. Each transmitter was attached to the posterior costal carapace with epoxy putty. No anesthetic or drug of any kind was used on the collected turtles in any moments during the study. Radios were attached to turtles in about two minutes. Radios did not harm or affect turtle behavior or health. Turtles were tracked with a Telonics TR–4 receptor and a four element (H) rubber ducky antenna. Once equipped with radios, turtles were tracked at least once per week from 2019 to 2021. Each time an individual was relocated in the field during telemetry surveys we recorded GPS location, microhabitat when inactive (leaf litter, woody debris, closest sarteneja, or other), macrohabitat (tall and short forest), and activity (walking, basking, courtship behavior, or foraging).

*Estimating home range size*. – Home range was estimated using two methods, the minimum convex polygon (MCP) and the Kernel density estimator (K; [31]). For the MCP estimate, we used 100% of the relocations in the home range estimate, but in the Kernel density estimators, we used 95% of the relocations in the estimation to minimize the influence of outliers and calculated the core home range area using 50% of the relocations to identify core use areas. A key detail about Kernel density estimators is that they require a smoothing parameter “h” to define the extent of the buffers that encapsulate the home range, and these can have a profound effect on home range size estimations. We calculated Kernel density estimations using three methods to determine “h”. The first is the default “href” estimate (Khref), the second is the Least Squared Cross Validation method (lscv; Klscv), and the third is a distance method that uses the average distances between relocations to define “h” (Kdistance; [32, 33]). We also calculated the home range overlap of the Kernel density estimate to quantify the amount of space species share. All home range analyses were performed with functions provided by the adehabitatHR [32] in R [34]. Home range maps were elaborated using ArcGIS 10.1 using Kdistance method [35].

*Estimating spatial overlap*. – To estimate spatial overlap between species and season we used the utilization distribution overlap index (UDOI) for each relocation [36]. This index is recommended for estimating the shared use of space among several species [37, 38]. UDOI values range from zero (no overlap) to 1 (uniformly distributed and have 100% overlap) but can be greater than 1 if both UDOI are nonuniformly distributed and have a high degree of overlap [36]. UDOI was estimated using adehabitatHR package [32] in the R environment [34].

*Relocation distances*. – Each time we recorded a turtle in the field we recorded its GPS location, allowing us to calculate the distance between consecutive relocations. We used these relocation distances to ask if relocation distances are correlated to season, straight-line carapace size, sex, and species. For distance moved by each tracked turtle, we used the UTM coordinates from each localization to calculate the following equation:

$$\sum _{i=1}^{n}=distance n \frac{\sqrt{{({e}_{2}-{e}_{1})}^{2}+({c}_{2}-{c}_{1}{)}^{2}}}{t}$$

When *e* is latitude in UTM, and *c* longitude in UTM and, *t* is the time between sampling events of coordinates *e*_*1*_, *c*_*1*_ with *e*_*2*_, *c*_*2*_. A correlation between monthly distance and precipitation was estimated using one-way ANOVAs. We used the Oxkutzcab weather station (Servicio Meteorológico Nacional) data for data on monthly precipitation.

*Data analysis*. – We use generalized linear models (GLM), generalized linear mixed models (GLMM), and general linear mixed models (LMM) models to test relationships (1) between home range size, sex, straight-line carapace length (SLC), and species with all data; (2) home range size, season, sex, and species after breaking up home ranges by season; and (3) relocation distance, season, sex, species, and SLC. Before analyzing data, we calculated the natural logarithm of dependent variables and assessed for normality, heteroscedasticity, and influence of potential nested factors such as individuals within species. Normality assumption was assessed using a Shapiro-Wilk test, heteroscedasticity was assessed visually by observing variation in residuals of variables of interest (e.g. Sex, SLC, Species), and influence of nested factors was assessed visually by looking at the distribution of residuals across potential nested factors (e.g. individuals within species). Preliminary analyses were done using lm() function in the R package *stats* [34], which assumes a normal distribution. When data were not normally distributed, we used alternative distributions (e.g. quasi-Poisson) using GLM and the glm() function in the stats package in R [34]. If nested factors were needed, we used LMM and GLMM with the lmer() function in the lme4 package in R [39]. To determine the significance of independent variables we performed backward stepwise regression using likelihood ratio tests (LRT) in the drop1() function in R [34]. The original model and the final model were compared to a null model using LRT in the anova() function [34]. The observed and relative frequencies were used to assess for potential differences in time of activity, microhabitat, and macrohabitat use.

## Results

*Radio telemetry effort*. – From November 2018 to August 2021 we found and placed radios on five *K. creaseri*, eight *R. areolata*, and eight *T. yucatana* for overlapping, but different time periods (Table 1). Of the individuals tracked, *T. yucatana* were the largest (x̄ = 147.1 mm), followed by *R. areolata* (x̄ = 135.3 mm), and *K. creaseri* (x̄ = 117.0 mm). The minimum number of days a turtle was tracked was 10 days, the maximum was 1078 days, and the average was 580.5 days (Table 1). Over the course of the study, we made 1824 observations on radio-tracked turtles, and 1370 of these observations were new turtle relocations. The number of relocations per individual ranged from 7 to 104 and averaged 65.2 relocations per individual.

**Table 1:** ID, dates tracked, number of observation and relocations, sex, and straight-line carapace length (SLC) of the tracked turtles in Yucatan. Individual 1004 was removed from species comparisons of home range. ID letter “K” corresponds to *Kinosternon creaseri*, “R” to *Rhinoclemmys areolata*, and “T” to *Terrapene yucatana.* * individuals not included in home range analysis

ID	Start Track	End Track	Number of observations	Number of Relocations	Sex	SLC
K_201	05/Nov/18	21/Sep/20	111	74	Male	115
K_4007	16/Nov/19	26/Jul/21	91	49	Male	112.7
K_4027	16/Nov/19	18/Oct/21	103	58	Female	107.3
K_4030	16/Nov/19	15/Sep/20	52	32	Female	101.1
K_4034	16/Nov/19	26/Jul/21	92	51	Female	149
R_1	05/Nov/18	03/Sep/20	110	98	Male	137.5
R_12	16/Nov/19	07/Jun/21	90	70	Male	129.5
R_15	16/Nov/19	26/Jul/21	96	77	Male	138
R_16*	15/Jun/21	30/Aug/21	9	8	Male	117.3
R_2	05/Nov/18	21/Sep/20	109	87	Male	130.3
R_21	21/Nov/19	18/Oct/21	85	64	Female	145.5
R_23	12/May/20	18/Oct/21	60	52	Female	150
R_6	05/Nov/18	21/Sep/20	109	100	Male	134.5
T_1001	05/Nov/18	21/Sep/20	116	95	Female	146.5
T_1002	05/Nov/18	21/Sep/20	121	101	Female	141.5
T_1003	16/Dec/19	18/Oct/21	83	53	Female	151.8
T_1004*	04/Sep/18	14/Sep/18	7	7	Female	149
T_1005	05/Nov/18	21/Sep/20	111	87	Male	134.8
T_1007	05/Nov/18	18/Oct/21	139	104	Male	152
T_1009	05/Nov/18	21/Sep/20	105	84	Female	147
T_1021*	16/Nov/19	07/Jul/20	25	19	Male	154

*Home range among species*. – The mean home range for all individuals of each species was similar for MCP, Khref, and Kdistance; but Klscv was substantially smaller (S1). We use MCP and Kdistance (Table 2) to interpret home range because there was less interindividual variation with these estimates, whereas Khref and Klscv appear to severely overestimate and underestimate individual home ranges, respectively (S1). *Terrapene yucatana #*1004 was removed from home range comparisons of all species in the full data set because this female was walking in a straight line toward the limits of the reserve, and we removed the radio to avoid losing it. For home range data we removed *R. areolata* #16 and *T. yucatana* #1021.

**Table 2:** Mean ± standard deviation of 100% minimum convex polygon (MCP), 95% kernel estimate with mean distance used for the h parameter (Kdistance), and 50% core areas of kernel density estimates using distance for h (K50distance). Units are hectares

Species	*n*	Total Relocations	MCP	Kdistance	K50distance
*Kinosternon creaseri*	5	264	35.9 ± 58.44	31.14 ± 23.75	4.6 ± 1.95
*Rhinoclemmys areolata*	7	556	33.5 ± 66.16	31.49 ± 38.16	5.48 ± 4.79
*Terrapene yucatana*	6	543	13.6 ± 16.51	24.65 ± 16.07	5.35 ± 2.96

*Spatial overlap among species*. – The three species studied have largely overlapping spatial patterns, but we did find some differences between seasons and species. During the dry season, the UDOI estimations showed overlap among all species that were not evenly distributed (Table 3), whereas during the wet season the UDOI estimations showed overlap among species except for *R. areolata* and *K. creaseri* that exhibited less spatial overlap (Table 4). The UDOI estimations of overlap looking at all data (both seasons) show that *R. areolata* and *K. creaseri* have high amounts of overlap, but *T. yucatana* has little overlap with both species, suggesting that *T. yucatana* does use distinct areas (Table 5).

**Table 3:** Measure of spatial overlap (UDOI) among three turtle species in the dry season. Values UDOI = 1 indicate a high spatial overlap, being greater than one when the species shows high overlap but are not evenly distributed

	*Rhinoclemmys aerolata*	*Terrapene yucatana*	*Kinosternon creaseri*
*Rhinoclemmys aerolata*	6.94		
*Terrapene yucatana*	1.98	19.73	
*Kinosternon creaseri*	6.98	17.1	2.83

**Table 4:** Measure of spatial overlap (UDOI) among three turtle species in rainy season. Values UDOI = 1 indicate a high spatial overlap, being greater than one when the species shows high overlap but are not evenly distributed

	*Rhinoclemmys aerolata*	*Terrapene yucatana*	*Kinosternon creaseri*
*Rhinoclemmys aerolata*	11.6		
*Terrapene yucatana*	1.56	7.75	
*Kinosternon creaseri*	0.96	1.4	23.8

**Table 5:** Measure of general of spatial overlap (UDOI) among three turtle species. Values UDOI = 1 indicate a high spatial overlap, being greater than one when the species shows high overlap but are not evenly distributed

	*Rhinoclemmys aerolata*	*Terrapene yucatana*	*Kinosternon creaseri*
*Rhinoclemmys aerolata*	4.88		
*Terrapene yucatana*	0.77	5.12	
*Kinosternon creaseri*	1.86	0.97	19.69

**Fig. 1:**
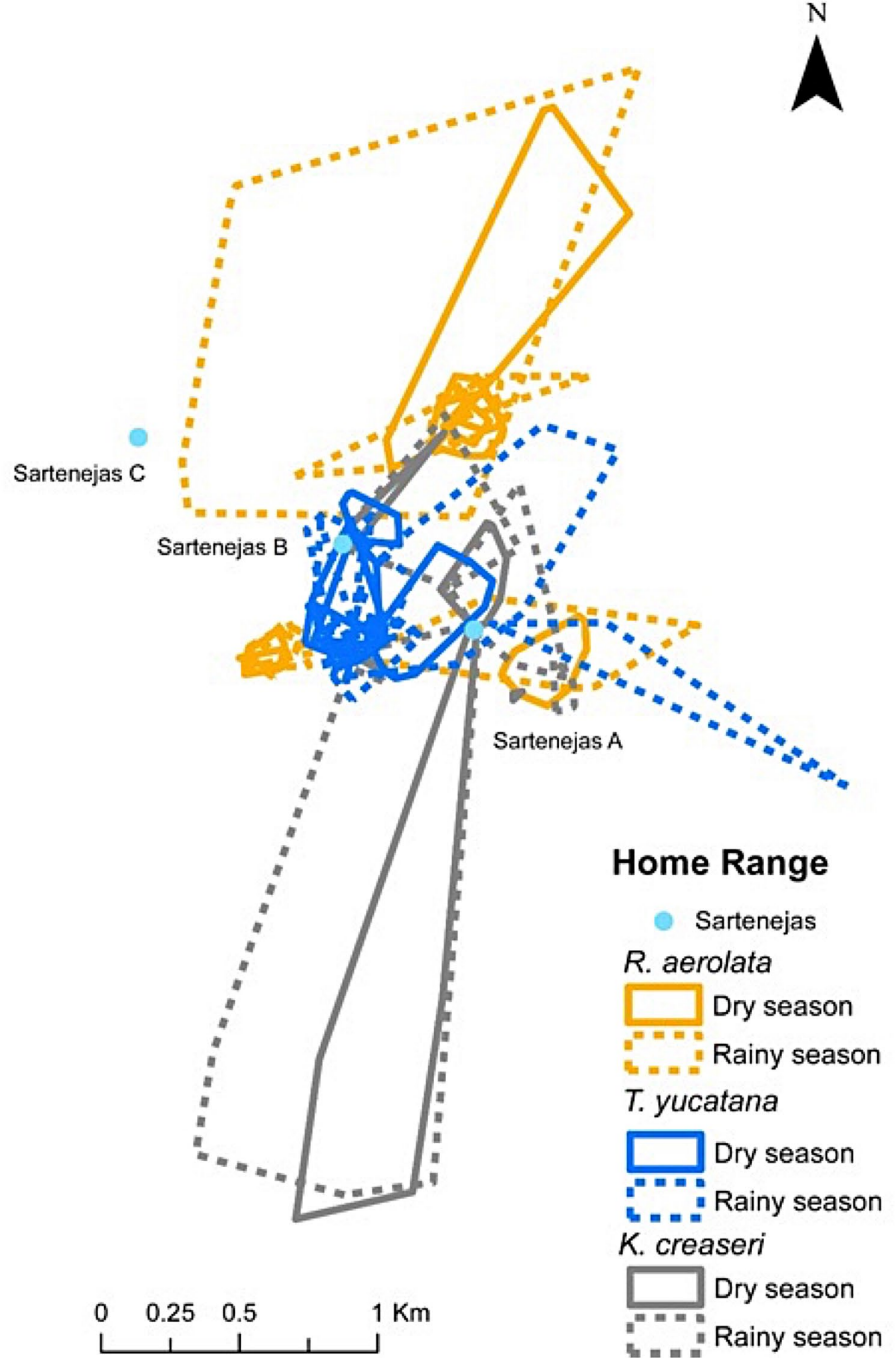
100% minimum convex polygons (MCP) home ranges for the 21 individuals belonging to three species that were monitored in this study. Color corresponds to species and each polygon represents the home range of an individual

Home range data were not normally distributed and followed an over-dispersed Poisson distribution. Of the three GLMM we fitted to test for differences in home range size, core home range, sex, species, and SLC we only found a significant difference between MCP size and Sex (Tables 2 and 6; Fig. 1). There was no significant relationship between Kdistance, SLC, season, or sex; and this was the same for K50distance, SLC, Season, or Sex (Table 6). Despite not exhibiting statistical significance, *K. creaseri* tended to have the largest home ranges, followed by *R. areolata* and *T. yucatana*, respectively (Table 6). When looking only at core area using K50distance, *R. areolata* had the biggest core area, followed by *T. yucatana*, and *K. creaseri* (Table 6).

**Table 6:** Results of generalized linear models (GLM) testing the fit between home range straight-line carapace length (SLC), Sex, and Species in all turtles with all data (1–3). Model 4 represents the results of the GLMM dividing home ranges by season testing the significance of home range size on season, species, and sex

Model number	Dependent Variable	Independent Variables	*n*	Scaled Deviance	*P* - value
1	MCP	SLC + Sex + Species	18	-5.45	0.24
		Sex		-3.58	0.046*
2	Kdistance	SLC + Sex + Species	18	-0.31	0.73
		no significant variables			
3	K50distance	SLC + Sex + Species	18	-0.01	0.78
		no significant variables			
4	Kdistance	Season + Species + Sex	82	-34.82	0.19
		Season		-25.54	0.04*

*Comparing home range between seasons*. – Separating the data by season resulted in data from four wet seasons and three dry seasons, representing the total of 82 home range estimates belonging to 19 turtles (S2). The distribution of these home ranges followed an over-dispersed Poisson distribution, and the GLMM was fitted using a quasi-Poisson distribution. Generalized linear mixed models’ results reveal a significant relationship between Kdistance home range and season, but not species or sex (Tables 6 and 7).

**Table 7:** Home range comparison between seasons in the three studied species. Data are in hectares. ± values are the standard deviation

Species	*n*	Season	Kdistance
*Kinosternon creaseri*	9	Dry	15.81 ± 9.24
	13	Rainy	16.87 ± 10.89
*Rhinoclemmys areolata*	13	Dry	15.17 ± 4.28
	18	Rainy	21.21 ± 15.96
*Terrapene yucatana*	13	Dry	13.04 ± 2.60
	16	Rainy	18.78 ± 10.03

*Distances Moved Between Relocations*. – There were 1370 relocations observed during the study, resulting in 1336 distances calculated between consecutive relocations, 34 relocations were less than 1 m and removed from further analysis. The average distance between relocations was 71.49 m, and the maximum distance moved between relocations was 1647 m for *K. creaseri*, 1058 m for *R. areolata*, 719 m for *T. yucatana*. Relocation data were normally distributed, but exhibited substantial variation among individuals within species, meriting the use of an LMM with turtle ID as a random effect nested within species. Results of LMM revealed significant differences in relocation distances between wet and dry seasons, but not between species or sex (Table 8). The average distance between relocations during the wet season was 93.3 m (± 149.9 m) and 48.8 m (± 98.5 m) during the dry season.

**Table 8:** Results from linear mixed model (LMM) testing the significance of Season, Sex, and Species on distances moved between relocations in *K. creaseri*, *R. areolata*, and *T. yucatana*. Full model and reduced models are indicated, *X*^2^ is the chi-square distributed likelihood ratio test (LRT) statistic, which differs from generalized linear models which used scaled deviance to perform LRT between models

Model	Dependent Variable	Independent variables	*n*	Random Effect	X^2^	*P* - value
Full	Distance	Season + Sex + Species	1336	Species: ID	121.59	< 0.0001
Reduced		Season			119	< 0.0001

All species tended to move more during the wet season. There was a significant relationship between average monthly rainfall and average monthly distance moved in *T. yucatana* (F = 18.43, *P* = 0.002) and *R. areolata* (F = 68.775, *P* < 0.001), but not in *K. creaseri* (F = 2.39, *P* = 0.153).

**Fig. 2:**
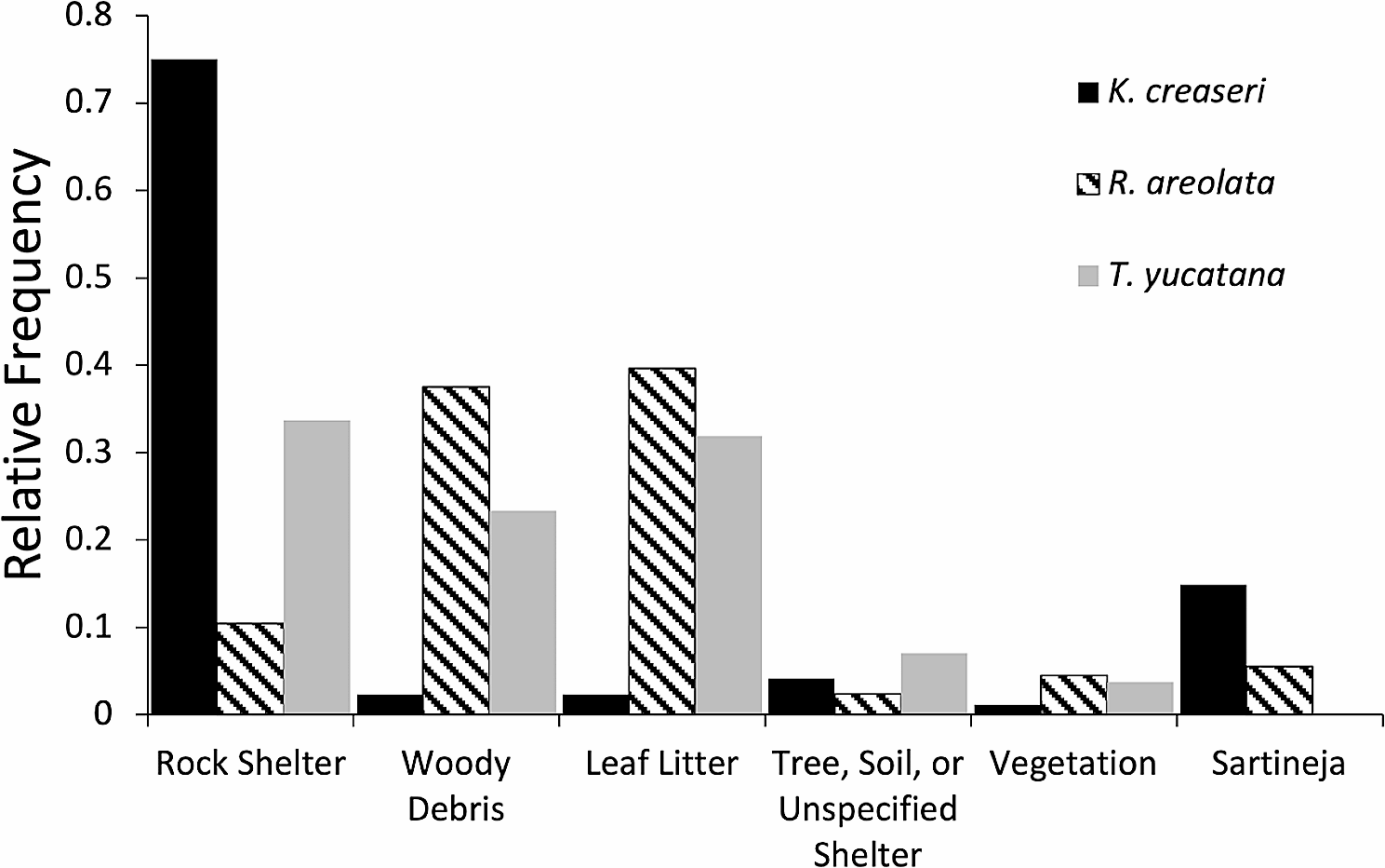
Relative frequency of microhabitats each species was observed using when encountered inactive in the field (*n* = 1241)

*Microhabitat of Inactive Turtles*. – Microhabitat of inactive turtles that were being monitored with radios was recorded during 1241 observations, microhabitats used include leaf litter, woody debris, vegetation (bromeliad, lianas, or other herbaceous vegetation), rock shelters, other types of habits (tree, soil, or unspecified), and sartenejas. *Kinosternon creaseri* was most frequently observed in rock shelters followed by sartenejas (Fig. 2). *Rhinoclemmys areolata* was observed inactive most frequently in woody debris and leaf litter (Fig. 2). *Terrapene yucatana* was observed most in rock shelters, leaf litter and woody debris (Fig. 2).

**Fig. 3:**
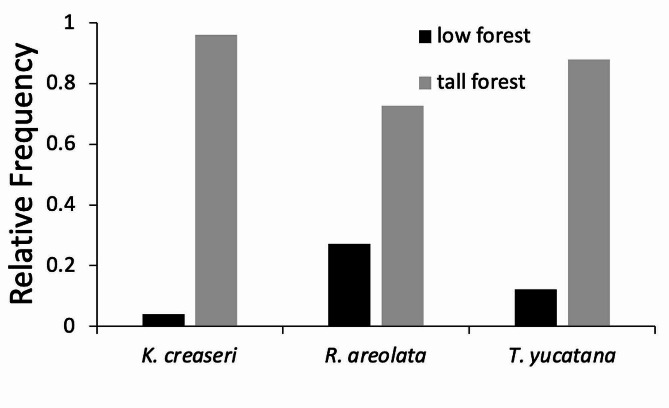
Relative frequency that each species in the study occurred in low or tall forest (*n* = 426)

**Fig. 4:**
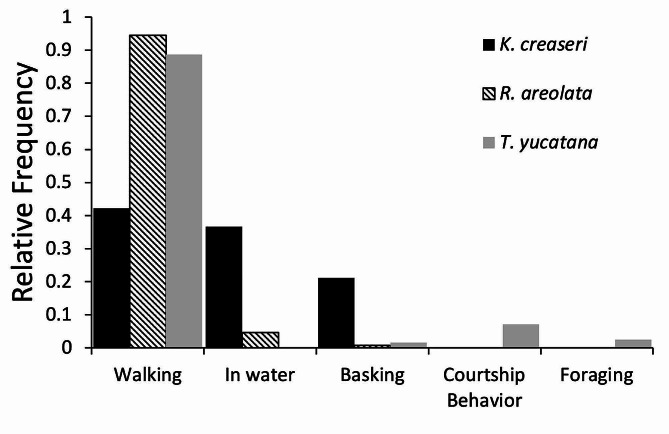
Relative frequency of different activities turtles was observed performing when encountered active in the field during radio telemetry surveys (*n* = 437)

*Macrohabitat and Behavior of Active Turtles*. – The macrohabitat of active turtles was recorded for 426/467 observations in which turtles were observed active. All turtles were observed most often in tall mature forest (Fig. 3), but *R. areolata* occurred in short forest more than the other species (Fig. 3). We recorded activity 437/467, and most of our observations consist of encountering turtles walking on the ground (Fig. 4). However, we also observed turtles in water, basking, in courtship behavior, and foraging (Fig. 4).

## Discussion

We provide the first comprehensive radio telemetry study of three sympatric turtle species in Yucatan, Mexico. Our findings reflected the distinct natural history of the three species, and we documented behaviors that demonstrate how these species can occupy similar landscapes but co-exist without competing for the same resources. We also found a significant impact of season on home range size and distances between relocations, with turtles having smaller home ranges during the dry season and moving shorter distances. We also documented key differences in microhabitat use, macrohabitat, and activity patterns that suggest that the three species we studied may partition habitat resources to minimize interspecific competition.

There was no significant difference in home range size among species, but with both MCP and Kdistance estimates *K. creaseri* had the largest home range size, followed by *R. areolata*, and *T. yucatana*. Nonetheless, most of *K. creaseri’s* home range size is comprised of areas that turtles were observed traversing to find sartenejas that are randomly dispersed throughout the landscape. The fact that *K. creaseri* has the largest home range, but smallest core area, reflects a key aspect of their natural history that is characterized by making large land movements to find small sartenejas across the landscape. When considering only the core area K50distance, *R. areolata* had the largest home range, followed by *T. yucatana*, then *K. creaseri*. Thus, our prediction that larger home ranges were associated to the biggest turtle, *R. areolata*, was partially fulfilled.

During the rainy season, home ranges were larger in all species studied compared to the dry season. Water availability and the resulting increase in resources are the main drivers that lead to turtles occupying larger home ranges during the wet season, a pattern that has been described for many animals living in seasonal habitats [40, 41]. During the rainy season, the smallest studied species *Kinosternon creaseri* had the largest home range, and its relocation data suggest that some individuals moved between one or two distinct sartenejas, and other individuals (e.g. 4007) traversed large distances apparently in search for other limestone pools. Sartenejas have been reported to be very important components for *K. creaseri* demography in Kaxil Kiuc [27]. During the dry season, individuals move to aestivate under rocks or small crevices, also some other individuals stay in the larger sartenejas that have water all year long. Aparicio et al. [42] reported a similar pattern in *K. integrum* from Michoacán. *Terrapene yucatana*, the larger species has the smallest home range and tend to exclusively inhabit the tallest and most humid parts of the forest (tall forest). *Rhinoclemmys areolata* seem to be habitat generalist, as they have the large core home range areas, they occupy short and tall forests, and they can take refuge in almost any kind of microhabitat. These observations coincide with previous reports of this species that have documented this species being a habitat generalist with an affinity to water [25]. During the dry season *R. areolata* reduces its home range and aestivates in diverse microhabitats. For our study site there are observations of *R. areolata* sharing the same habitat with *K. creaseri* [25, Butterfield direct obs.].

According with Slavenko et al. [28], body size is one of the main drivers to explain home range due the energetic requirements of turtles. Our data partially agree with Slavenko et al. [28], being that the *Rhinoclemmys areolata*, which are generally larger turtles than the other species in this study, had large core home range sizes, but since the more aquatic (or less terrestrial) species (*K. creaseri*) also was the one with the second largest home range of the studied species. The more terrestrial species have sporadic aquatic incursions only during the rainy season, and these tend to be in ephemeral puddles following a rain, and *R. areolata* was rarely seen in bigger sartenejas. During dry season the three species limited their activity, and this pattern of home range reduction during the dry season has been reported elsewhere in tropical and subtropical systems [17, 43, 44].

It is clear from the home range and distance data that the activity patterns of these turtles are coupled with water availability, as distances between relocations were correlated with the increase of precipitation during the rainy season. Similar patterns of seasonality have been observed in other turtles from the dry tropics [17, 42, 43, 45, 46]. All turtles in our study moved less during the dry season and despite many sartenejas maintaining water throughout the dry season, most turtles estivate and spend their time in microhabitats such as rock shelters, leaf litter, and woody debris between December - May. *Kinosternon creaseri* used almost exclusively rock shelters as microhabitats when inactive, *R. areolata* used mainly woody debris and leaf litter, and *T. yucatana* used nearly similar proportions of rock shelters, woody debris, and leaf litter when inactive.

During the rainy season freshwater turtles mate and nest [47], as well as feed and store energy reserves for the next dry season and aestivation period [48, 49]. As predicted, the three studied species moved longer distances during rainy season and were observed walking, in courtship, foraging, and basking. When active a disproportionate number of our observations were comprised of turtles walking on land, only *K. creaseri* spends significant time in water, with *R. areolata* only rarely occurs in water.

As detected in other studies [28], our estimate of MCP tend to overestimate home range, since is very sensitive to outliers and a small number of relocalizations. On the other hand, our 95% K (and the estimated core home range) showed smaller home ranges but with the same pattern of MCP. Similar results were described in Enríquez-Mercado et al. [45] and Aparicio et al. [42] when both methods were used. Jones et al. [46] estimated a 0.68 ha home range for *T. yucatana* in northern Yucatan using MCP (adjusted at 95%), which falls within our estimated range for dry and rainy season of when we used 95% K, but did not match with our MCP estimate for Kaxil Kiuc, however Jones et al. [46] study had a larger sample size for a longer period of time for their estimation, nevertheless that study site lacks of permanent ponds or sartenejas, been the typical northern Yucatan Peninsula landscape without superficial bodies of water.

## Conclusions

Our study highlights key similarities and differences in how turtles use space and habitats on the Yucatan peninsula. A unique aspect of the Yucatan peninsula is that there are no rivers, and the only water bodies on the landscape are where water accumulates during the wet season such as sartenejas, or where there is permanent water in cenotes. In our study, there were only three large sartenejas wider than 2 m in diameter, the rest that were observed were less than 1 m. While *T. yucatana* and *R. areolata* do not depend on water bodies, *K. creaseri* relies on water to mate and forage and our data show that *K. creaseri* overcomes this by traversing long distances between water bodies. We also find that *R. areolata* seems to be distributed in different forest successional stages, with *T. yucatana* mainly being part of mature tall forest, and *R. areolata* being found in similar proportions in all habitats. Together these data suggest that the three species of Yucatan partition resources, with *K. creaseri* relying on sartenejas, *R. areolata* being a semi- terrestrial species with a generalist to habitats it occurs in, and *T. yucatana* exclusively terrestrial and tending to occur in mature forests.

### Electronic supplementary material

Below is the link to the electronic supplementary material.

Supplementary Material 1

## Data Availability

The datasets generated and analyzed during the current study are available in the following Dropbox link: https://www.dropbox.com/scl/fo/k250zuz3cnvweiwnqbft9/h?rlkey=qj81xnhtsqs9lpjtmdc49ua4o&dl=0.
