# Supplementary material for: Home range of three turtle species in Central Yucatan. A comparative study
Source: BMC Ecol Evol. 2024 May 29;24:71. doi: 10.1186/s12862-024-02258-7 (PMC11134725; doi:10.1186/s12862-024-02258-7)
Supplement: Supplementary file 1 — Supplementary Material 1 [file 12862_2024_2258_MOESM1_ESM.docx]

Supplementary material

S1. “href” and LSCV values for all the tracked turtles. ID letter “K” corresponds to *Kinosternon creaseri*, “R” to *Rhinoclemmys areolata*, and “T” to *Terrapene yucatana.*

| ID | Khref | Average (± SD) | Klscv | Average (± SD) |
| --- | --- | --- | --- | --- |
| K_201 | 26.84 | 116.59 (± 228.28) | 1.06 | 5.35 (± 10.16) |
| K_4007 | 524.55 |  | 23.52 |  |
| K_4027 | 1.37 |  | 0.24 |  |
| K_4030 | 7.96 |  | 0.66 |  |
| K_4034 | 22.26 |  | 1.27 |  |
| R_1 | 5.86 | 50.06 (± 117.37) | 2.32 | 6.61 (± 7.97) |
| R_12 | 4.35 |  | 2.16 |  |
| R_15 | 6.08 |  | 4.58 |  |
| R_16 | 9.97 |  | 2.70 |  |
| R_2 | 340.21 |  | 25.49 |  |
| R_21 | 9.53 |  | 9.17 |  |
| R_23 | 3.06 |  | 2.14 |  |
| R_6 | 21.46 |  | 4.37 |  |
| T_1001 | 22.39 | 43 (± 69.50) | 1.71 | 11.63 (± 23.33) |
| T_1002 | 1.82 |  | 0.63 |  |
| T_1003 | 81.20 |  | 9.85 |  |
| T_1004 | 204.01 |  | 76.37 |  |
| T_1005 | 5.33 |  | 0.55 |  |
| T_1007 | 12.67 |  | 1.63 |  |
| T_1009 | 5.88 |  | 0.96 |  |
| T_1021 | 18.42 |  | 1.37 |  |

S2. A total of 85 home range estimates belonging to 21 turtles tracked in the study. ID corresponds to species and individual with the letters corresponding to the species, and the numbers to the individuals. SLC = straight line carapace, season corresponds to wet season (June-October), dry season (November – May). Kdistance corresponds to the home range calculation for each partition of the data, data are in hectares (ha). is the ID letter “K” corresponding to *Kinosternon creaseri*, “R” to *Rhinoclemmys areolata*, and “T” to *Terrapene yucatana.*

| ID | Sex | SLC | Season | Kdistance (ha) |
| --- | --- | --- | --- | --- |
| K_201 | Male | 115 | Wet | 9.888826 |
| K_201 | Male | 115 | Dry | 10.400346 |
| K_201 | Male | 115 | Dry | 13.91481 |
| K_201 | Male | 115 | Wet | 13.95773 |
| K_201 | Male | 115 | Wet | 18.14382 |
| K_4007 | Male | 112.7 | Dry | 10.179048 |
| K_4007 | Male | 112.7 | Wet | 12.63386 |
| K_4007 | Male | 112.7 | Dry | 38.4633 |
| K_4007 | Male | 112.7 | Wet | 50.44647 |
| K_4027 | Female | 107.3 | Dry | 10.42068 |
| K_4027 | Female | 107.3 | Wet | 10.66256 |
| K_4027 | Female | 107.3 | Wet | 10.83423 |
| K_4027 | Female | 107.3 | Wet | 10.88749 |
| K_4027 | Female | 107.3 | Dry | 11.213325 |
| K_4030 | Female | 101.1 | Dry | 9.851089 |
| K_4030 | Female | 101.1 | Wet | 11.63905 |
| K_4030 | Female | 101.1 | Wet | 12.60432 |
| K_4034 | Female | 149 | Wet | 15.06413 |
| K_4034 | Female | 149 | Wet | 17.97153 |
| K_4034 | Female | 149 | Dry | 18.40851 |
| K_4034 | Female | 149 | Dry | 19.49034 |
| K_4034 | Female | 149 | Wet | 24.60134 |
| R_1 | Male | 137.5 | Wet | 10.747801 |
| R_1 | Male | 137.5 | Dry | 10.87054 |
| R_1 | Male | 137.5 | Dry | 12.306101 |
| R_1 | Male | 137.5 | Wet | 12.88564 |
| R_1 | Male | 137.5 | Wet | 27.48938 |
| R_12 | Male | 129.5 | Dry | 11.715654 |
| R_12 | Male | 129.5 | Dry | 12.7112 |
| R_12 | Male | 129.5 | Wet | 14.41639 |
| R_12 | Male | 129.5 | Wet | 14.90756 |
| R_15 | Male | 138 | Wet | 12.24366 |
| R_15 | Male | 138 | Wet | 12.64186 |
| R_15 | Male | 138 | Dry | 12.727788 |
| R_15 | Male | 138 | Dry | 14.72687 |
| R_15 | Male | 138 | Wet | 21.16337 |
| R_16 | Male | 117.3 | Wet | 16.28123 |
| R_2 | Male | 130.3 | Wet | 14.550019 |
| R_2 | Male | 130.3 | Dry | 17.406548 |
| R_2 | Male | 130.3 | Dry | 26.323943 |
| R_2 | Male | 130.3 | Wet | 38.75289 |
| R_2 | Male | 130.3 | Wet | 76.71921 |
| R_21 | Female | 145.5 | Dry | 16.003258 |
| R_21 | Female | 145.5 | Dry | 16.3538 |
| R_21 | Female | 145.5 | Wet | 16.67984 |
| R_21 | Female | 145.5 | Wet | 16.9514 |
| R_23 | Female | 150 | Dry | 10.75641 |
| R_23 | Female | 150 | Wet | 11.32395 |
| R_23 | Female | 150 | Wet | 12.23699 |
| R_6 | Male | 134.5 | Wet | 11.13193 |
| R_6 | Male | 134.5 | Dry | 15.916053 |
| R_6 | Male | 134.5 | Dry | 19.329928 |
| R_6 | Male | 134.5 | Wet | 25.57526 |
| R_6 | Male | 134.5 | Wet | 31.3105 |
| T_1001 | Female | 146.5 | Dry | 12.148727 |
| T_1001 | Female | 146.5 | Dry | 17.017318 |
| T_1001 | Female | 146.5 | Wet | 18.312412 |
| T_1001 | Female | 146.5 | Wet | 20.77831 |
| T_1001 | Female | 146.5 | Wet | 26.13141 |
| T_1002 | Female | 141.5 | Wet | 9.863085 |
| T_1002 | Female | 141.5 | Dry | 10.106085 |
| T_1002 | Female | 141.5 | Dry | 10.306769 |
| T_1002 | Female | 141.5 | Wet | 11.67758 |
| T_1002 | Female | 141.5 | Wet | 15.02603 |
| T_1003 | Female | 151.8 | Dry | 12.903531 |
| T_1003 | Female | 151.8 | Dry | 14.87131 |
| T_1003 | Female | 151.8 | Wet | 35.64783 |
| T_1003 | Female | 151.8 | Wet | 48.61428 |
| T_1004 | Female | 149 | Wet | 39.323084 |
| T_1005 | Male | 134.8 | Dry | 9.971813 |
| T_1005 | Male | 134.8 | Dry | 10.264671 |
| T_1005 | Male | 134.8 | Wet | 13.32933 |
| T_1005 | Male | 134.8 | Wet | 14.730365 |
| T_1005 | Male | 134.8 | Wet | 18.91639 |
| T_1007 | Male | 152 | Wet | 10.2342 |
| T_1007 | Male | 152 | Dry | 10.53208 |
| T_1007 | Male | 152 | Wet | 11.9832 |
| T_1007 | Male | 152 | Dry | 15.146239 |
| T_1007 | Male | 152 | Dry | 15.620662 |
| T_1007 | Male | 152 | Wet | 17.3496 |
| T_1009 | Female | 147 | Wet | 13.00023 |
| T_1009 | Female | 147 | Dry | 14.861721 |
| T_1009 | Female | 147 | Wet | 14.9619 |
| T_1009 | Female | 147 | Dry | 15.72116 |
| T_1021 | Male | 154 | Dry | 14.86977 |
